# Flavor Characterization of Native Xinjiang Flat Peaches Based on Constructing Aroma Fingerprinting and Stoichiometry Analysis

**DOI:** 10.3390/foods12132554

**Published:** 2023-06-29

**Authors:** Chunyan Li, Youyou Xu, Huimin Wu, Ruirui Zhao, Xinwei Wang, Fangfang Wang, Qingquan Fu, Tiantian Tang, Xuewei Shi, Bin Wang

**Affiliations:** Food College, Shihezi University, Shihezi 832000, China; 18892996689@163.com (C.L.); xuyouyou2513@163.com (Y.X.); wu13834139594@163.com (H.W.); z17699534005@163.com (R.Z.); wxw990610@163.com (X.W.); wff200905@163.com (F.W.); fu19915238669@163.com (Q.F.); 15124534465@163.com (T.T.); shixuewei@shzu.edu.cn (X.S.)

**Keywords:** flat peach (*Prunus persica* L. *Batsch. var. compressa Bean*), phenolic compounds, antioxidant activity, aroma fingerprinting, stoichiometry analysis

## Abstract

The flat peach is a high economic value table fruit possessing excellent quality and a unique aroma. This article investigated the quality characteristics and aroma fingerprinting of flat peaches (Qingpan, QP; Ruipan 2, R2; Ruipan 4, R4; Wanpan, WP) from Xinjiang in terms of taste, antioxidant capacity, and volatile aroma compounds using high-performance liquid chromatography (HPLC) and HS-SPME-GC-MS. The results showed that the flat peaches had a good taste and high antioxidant capacity, mainly due to the high sugar–low acid property and high levels of phenolic compounds. This study found that sucrose (63.86~73.86%) was the main sugar, and malic acid (5.93~14.96%) and quinic acid (5.25~15.01%) were the main organic acids. Furthermore, chlorogenic acid (main phenolic compound), epicatechin, rutin, catechin, proanthocyanidin B1, and neochlorogenic acid were positively related to the antioxidant activity of flat peaches. All flat peaches had similar aroma characteristics and were rich in aromatic content. Aldehydes (especially benzaldehyde and 2-hexenal) and esters were the main volatile compounds. The aroma fingerprinting of flat peaches consisted of hexanal, 2-hexenal, nonanal, decanal, benzaldehyde, 2,4-decadienal, dihydro-β-ionone, 6-pentylpyran-2-one, 2-hexenyl acetate, ethyl caprylate, γ-decalactone, and theaspirane, with a “peach-like”, “fruit”, and “coconut-like” aroma. Among them, 2,4-decadienal, 2-hexenyl acetate, and theaspirane were the characteristic aroma compounds of flat peaches. The results provide a theoretical basis for the industrial application of the special aroma of flat peaches.

## 1. Introduction

The peach (*Prunus persica*), belonging to the Rosaceae family, is widely cultivated in 80 areas and nations around the world, such as America, Japan, and China [1]. The flat peach (*P. persica* L. *Batsch. var. compressa Bean*), a kind of peach like a saucer or doughnut, originated in the Xinjiang region in the northwest of China and has been cultivated for over 2000 years [2,3]. Flat peaches are widely grown in Xinjiang, mainly in the Shihezi, Usu, Kashgar, and Yili regions [4]. What is more important is that the “the flat peach of 143rd regiment in Shihezi” has been officially approved by the Ministry of Agriculture and Rural Affairs of the People’s Republic of China for registration and protection of geographical indications for agricultural products (http://www.moa.gov.cn/govpublic/ncpzlaq/201704/t20170424_5580538.htm, accessed on 20 April 2017). Xinjiang has a typically temperate and continental climate with ample sunlight, a massive temperature distinction between day and night, and lengthy sunshine hours, which endue the excellent quality of flat peaches [5]. Because of its sweetness, taste, and charming fruit aroma, flat peaches have become a popular table fruit in Xinjiang and are sold all around the world [6].

The acceptance of consumers plays an important role in the judgment of fruit quality. Organoleptic attributes (including SSC and TA, texture) and qualitative attributes (including sweetness and acidity) are important indicators for consumers to evaluate the traits of the fruit [7]. Taste is the first impression the fruit gives to the consumer. As a well-known table fruit, the flat peach possesses juicy flesh, a non-melting texture, and high sugar–low acid properties, which endue the flat peach with a good mouthfeel [8]. Sweetness and acidity are also among the most important quality indicators of the fruit’s appeal to consumers. There is some research suggesting that flat peaches contain high levels of organic acids (especially malic acid, quininic acid, and citric acid) [9,10] and soluble sugars (including sucrose, fructose, and glucose) [11]. In addition to its organoleptic and qualitative properties, the flat peach is endowed with functional properties due to the presence of amino acids, vitamin C, and high levels of antioxidant substances (especially phenolic compounds) and other bioactivators [12]. Modern clinical studies have proven that phenolic compounds and their meal substrates have anti-proliferative, anti-inflammatory, immunomodulatory, cardiac, neurological, and liver-shielding effects on the human body [13]. It is proven that the major phenolic compounds in the flat peach are usually procyanidin and quercetin, and these compounds are associated with antioxidant potential [14,15,16].

Apart from the inherent taste, texture, and nutritional properties of the fruit, the aroma is also an important quality characteristic [17]. Fruits often have their own distinctive and typical aroma characteristics. With headspace solid-phase microextraction combined with gas chromatography–mass spectrometry (HS-SPME-GC-MS) being widely used for the detection of volatile compounds in various types of fruit, more and more aroma substances that contribute significantly to the odor of peaches are being determined [18,19]. Until now, over 100 volatile compounds in peach fruits have been identified [20]. It is believed that the flavor compounds of peaches consist of esters, lactones, aldehydes, alcohols, and ketones [21,22], especially heptanal, 2-hexenal, and octanal [23]. These compounds give the peach a distinct “peach-like” and “fresh and grassy” flavor [24]. Studies have shown that the flavors of peach fruits are relatively similar, but there are differences between varieties. The white-fleshed peach has been reported to be rich in (E)-2-nonenal, 1-pentanol, and styrene, while the yellow-fleshed peach has significant amounts of (Z)-3-hexenyl acetate, n-octanal, pelargonaldehyde, and 3,5-octadien-2-one [25]. Hexanol, hexanal, benzaldehyde, and cis-3-hexenyl acetate are considered to be the main aroma substances affecting the honey peach [26]. In contrast, nectarines are rich in C13-norisoprenoids (mainly hydroxy-7,8-dihydro- β-ionone, and 4,5-dehydrovomifoliol) [27]. Of note, only some volatile compounds have an important influence on the aroma of the flat peach. Odor activity values (OAVs) are therefore used to identify volatile compounds that contribute significantly to the odor characteristics of flat peach [28,29].

In summary, the peach has a typical aroma, but there are significant differences between varieties. Although Tan Fengling et al. (2022) determined the composition of volatile aroma compounds in flat peach juice [30], the quality characteristics and aroma fingerprinting of the flat peach have not been reported. Based on the above, the aim of this study was to investigate the quality characteristics and aroma fingerprinting of flat peaches from Xinjiang in terms of taste, antioxidant capacity, and volatile aroma compounds using high-performance liquid chromatography (HPLC) and HS-SPME-GC-MS. The contents of soluble sugars, organic acids, and phenolic compounds in the flat peach were identified by HPLC, and the correlation between phenolic compounds and antioxidant activity is discussed. HS-SPME-GC-MS was used to determine the composition and content of volatile aroma compounds of the flat peach. Principal component analysis (PCA) was used for discriminating unbiased variations and aroma biomarkers of the flat peach; then, multivariate statistics were combined to construct the aroma fingerprinting of flat peaches from Xinjiang. This study can provide a more comprehensive understanding of the characteristic qualities and aroma of the flat peach and lay a theoretical basis for cultivating the aroma, nutritional value, and medicinal value of the flat peach. In addition, the present results will be of outstanding significance in the industrial manufacture of the special aroma of flat peaches from Xinjiang.

## 2. Materials and Methods

### 2.1. Samples

**Figure 1:**
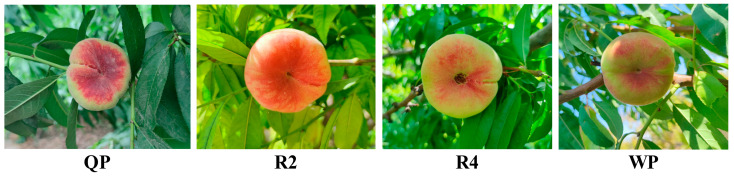
Flat peaches used in the present experiment. Qingpan (QP), Ruipan 2 (R2), Ruipan 4 (R4), Wanpan (WP) from Shihezi, Xinjiang.

Flat peaches (Qingpan, QP; Ruipan 2, R2; Ruipan 4, R4; Wanpan, WP) were obtained at the commercial maturity stage from 6-year-old flat peach trees in Shihezi, Xinjiang, China (44°29″ N, 89°03″ E) (Figure 1). Sixty fruits of each flat peach were picked at different locations in the orchard, all of which were randomly divided into three replicates, for a total of 240 fruits. All flat peaches were non-damaged and non-rotten with a crisp texture (firmness greater than 9 N) (Supplementary Table S1). These samples were transported to the laboratory at once, washed with distilled water within 2 h, and then stored in sealed polyethylene bags at −4 ℃ for subsequent analytical experiments.

### 2.2. Chemicals

Controls, consisting of sugars (fructose, sorbitol, glucose, sucrose), organic acids (quinic acid, malic acid, citric acid, succinic acid), phenolic compounds (coumaric acid, procyanidin B1, neochlorogenic acid, catechin, vanillic acid, chlorogenic acid, epicatechin, rutin, and quercetin), and 3-octanol were bought from Shanghaiyuanye Bio-Technology Co., Ltd. (Shanghai, China). The reagents for 2,2’-azinobis (3-ethylbenzothiazoline-6-sulphonate) (ABTS^+^), 2,2-diphenyl-1-picrylhydrazyl (DPPH), ferric reducing antioxidant power (FRAP), and cupric reducing antioxidant capacity (CUPRAC) were bought from TANEE CHEMICAL LTD. Analytical-grade reagents were bought from business sources for the remaining materials.

### 2.3. Determination of Soluble Solid Content (SSC) and Titratable Acidity (TA)

The SSC of the flat peaches was determined in accordance with the approach of Deena Ramful et al. (2011) via the utilization of a sugar refractometer (Atago PR-101R, Tokyo, Japan) after pressing and filtering the fruit residues [31]. Referring to Mohd Fadzelly Abu Bakar et al. (2009)’s method, with some improvements, the TA was determined by titration with NaOH (0.1 mol/L), and the expression of the results was the percentage (%) of malic acid [32]. Each replicate contained 10 fruits and all assays were carried out in triplicate.

### 2.4. Determination of Total Phenol Content (TPC) and Total Flavonoid Content (TFC)

The TPC of the samples was estimated in accordance with the Folin–Ciocalteu method [33]. In order to fully extract the phenolic compounds in flat peaches, the samples were pretreated by freeze-drying for 24 h in a vacuum freeze-dryer (Christ Alpha 2–4, Osterode, Germany) before being ground in a mortar and pestle to obtain lentil powder. An amount of 2.5 g of lyophilized trituration and 12.5 mL of 80% (*v*/*v*) aqueous methanol were placed as a mixture in a capped centrifuge tube and extracted by sonication at 35 °C for 30 min. The tube was centrifuged by 10,000 rotations per minute (rpm) for 20 min after ultrasound extraction. After repeating the above operation 2 times, the supernatant was combined and used as a sample solution for the next determination. An amount of 1 mL of the above extract was mixed with an equal amount of 5-times diluted Folin-Ciocalteu reagent, then put into a tube and left to stand for 5 min. Next, 4 mL of Na_2_CO_3_ (7.5%; *w*/*w*) was added and left to react for 60 min at room temperature in the dark. Absorbance values were measured at 765 nm using an ultraviolet spectrophotometer. Gallic acid (GA) was chosen as the standard, and TPC was expressed in mg of gallic acid equivalents of fresh fruit (mg GAE/100 g FW).

The determination of TFC was based totally on the preceding technique with some modifications [34]. An amount of 1 mL of polyphenolic extract was added to 0.1 mL of NaNO_2_ solution (5%; *w*/*w*) in a tube. The tube with the mixture was left to stand for 5 min, 0.15 mL of AlCl_3_·6H_2_O solution (10%; *w*/*w*) and 0.6 mL of NaOH solution (0.1 mol/L) were added successively at 5 min intervals and allowed to mix, and then distilled water was added to fix the volume to 3 mL. The absorbance was measured at 510 nm using an ultraviolet spectrophotometer. Rutin (RE) was chosen as the control substance and the results were expressed as mg of rutin equivalents in 100 g (mg RE/100 g FW).

### 2.5. Determination of Soluble Sugars and Organic Acids by HPLC

Soluble sugars and organic acids were extracted as described by Primoz Orazem et al. (2011), with some modifications [35]. Grind the pitted pulp tissue, weigh 1.0 g of the pulp, mix it with 20 mL of distilled water, and place it in a centrifuge tube. Use a vortexer to homogenize the mixture for 1 min, sonicate it for 30 min at 30 °C, and centrifuge it at 10,000 rpm for 15 min at 4 °C. Repeat the vortex extraction twice, take the supernatant and mix it into an extract, fill it to 40 mL, and subsequently filter it with a 0.22 μm water filter.

The methods of Haejin Bae et al. (2014) [36] and Christophe Aubert et al. (2014) [37], respectively, were used to estimate the content of soluble sugars and organic acids in flat peaches, combined with an HPLC system (Agilent 1200, Palo Alto, CA, USA). Of note, the detection of soluble sugars requires the combination of the refractive index detector-10A (RID-10A, Agilent Technologies 112 Inc, Palo Alto, CA, USA) differential detector for the elution peaks. For the determination of soluble sugar contents, acetonitrile, and water (80:20, *v*/*v*) as a mobile phase, the soluble sugar of the samples was separated by an XB ridge amide column (5 μm, 4.6 × 250 mm, Agilent, Santa Clara, CA, USA). For the determination of organic acid, a Waters C18 column (5 μm, 4.6 × 150 mm, Waters, MA, USA) was used for separation, 3% methanol was used as liquid A, and ultrapure water (pH 2.0) was used as liquid B. Liquid A and liquid B are collectively referred to as the mobile phase. Organic acids were measured at 210 nm with three replicates per sample. The flow rate was set to 0.8 mL/min, the injection volume was 10 μL, and the column temperature was 30 °C, according to the isocratic elution method.

### 2.6. Determination of Phenolic Compounds by HPLC

In order to identify the phenolic compounds in flat peaches it is first necessary to extract the phenolic compounds from the flesh of the fruit. We used the technique of Chang Susan et al. (2000), with some modifications [38]. An amount of 3 g of lyophilized powder and 15 mL of 80% (*v*/*v*) aqueous methanol were placed as a mixture in a capped centrifuge tube, vortexed for 1 min with an 80% (*v*/*v*) methanol–ultrapure water mixture to a total of 30 mL, and then stored at −4 °C until analysis.

Phenolic compounds (coumaric acid, procyanidin B1, neochlorogenic acid, catechin, vanillic acid, chlorogenic acid, epicatechin, rutin, and quercetin) were determined by HPLC in combination with a phase alignment detector (PDA, Waters, MA, USA). We used the Waters C18 column (5 μm, 4.6 × 150 mm; Waters, MA, USA). The injection volume was set at 10 μL and the flow rate was 0.8 mL/min. The column temperature was maintained at 30 °C. The mobile phase was methanol (A) and a 1% concentration of acetic acid in water (B). The elution was a gradient elution with the following elution procedures: 5% A at 0 min, 40% A at 35 min, 95% A at 55 min, and 5% A at 60 min. In accordance with Isabel Juániz et al. (2016) [39], the chlorogenic acid and neochlorogenic acid were evaluated at 330 nm; coumaric acid, procyanidin B1, catechins, vanillic acid, and epicatechin were evaluated at 280 nm; and rutin and quercetin were evaluated at 360 nm. The results of the phenolic compounds were expressed in mg/g FW.

### 2.7. Determination of Antioxidant Activity In Vitro

With reference to the previous approach and some modifications, the 2,2′-azinobis (3-ethylbenzothiazoline-6-sulfonic acid) (ABTS^+^) radical scavenging assay [40] and 2,2-Diphenyl-1-picrylhydrazyl (DPPH) radical scavenging rate [41] were evaluated in turn. The ferric reducing antioxidant power (FRAP) assay [41] and cupric ion (Cu^2+^) reducing (CUPRAC) assay [42] were also measured. It is really worth noting that when measuring the ABTS^+^ radical scavenging assay, 0.1 mL of polyphenolic extract and 3.9 mL of ABTS^+^ solution were reacted in the dark for 8 min, and the absorbance was measured at 732 nm. When measuring the DPPH radical scavenging rate, 0.1 mL of polyphenol extract and 3.9 mL of DPPH solution were reacted for 30 min under darkish conditions, and the absorbance was measured at 517 nm. All results are expressed as μmol Trolox equivalents in 100 g of fresh flat peach (μmol TEs/100 g FW).

### 2.8. Determination of Volatile Aroma Compounds by HS-SPME-GC-MS

With reference to the modified approach of Zhou Huijuan et al. (2018), the HS-SMPE-GC-MS method was used to determine the accumulation of volatile compounds in the flat peaches [43]. An amount of 4.5 g of lyophilized powder and 0.5 g of NaCl were loaded into a 20 mL headspace vial, then 2 μL of 330 μg/kg 3-octanol solution was added as the internal standard. Next, in a magnetic stirrer (HS-40, IKA, Germany), the capped headspace vial to which the sample to be measured was added was equilibrated at 45 °C and 300 r/min for 15 min using a magnetic rotor. The extraction SPME fibers, which were activated at 250 °C for 2 h, were selected from a divinylbenzene/carboxyl/polydimethylsiloxane (DVB/CAR/PDMS, 50/30 μm, StableFlex, Supelco, Bellefonte, PA, USA) extraction head. Finally, the extraction head was inserted into a headspace vial at 40 °C for 45 min at 300 rpm.

The volatile compounds in the flat peaches were evaluated by the Agilent 7000D-GC-MS detector (Agilent Technologies Inc., Santa Clara, CA, USA) and separated by the HP-Innowax column (30 m × 0.25 mm × 0.25 µm, Agilent Technologies Inc., Santa Clara, CA, USA). The extraction head was desorbed in the GC feeder for 7 min at 250 °C in non-split mode after the extraction operation. The column oven temperature was set to 230 °C in non-split injection mode. Helium was selected as the mobile phase and the ramp-up procedure was initiated at a constant flow rate of 1 mL/min, using 40 °C as the initial temperature, and held for 5 min. The temperature was then steeply increased to 86 °C at 4 °C/min and held for 5 min, then to 90 °C at 1.5 °C/min, to 180 °C at 5 °C/min and held for 3 min, and then steeply increased to 230 °C at 10 °C/min and held at 230 °C for 2 min before cooling back to 40 °C. Total ion chromatograms (TICs) were generated using an electron impact (EI) ionization mode with an ionization energy of 70 eV, mass spectrometry scan conditions set to a range of 35 to 350 m/z, a speed of 5 scans per second, and an ionization source temperature set to 270 °C.

The chromatograms of the isolated compounds were compared using the NIST spectral library (NIST 14, National Institute of Standards and Technology, Gaithersburg, MD, USA) and combined with the mass spectral library of Wiley 6 for the preliminary identification of volatile compounds in the flat peaches. Only identified compounds with a threshold match of more than 85% were selected. To improve the accuracy of substance matching, the retention time (RT) was also compared with its associated retention index (RI). A semi-quantitative method was used to calculate the relative concentrations of the volatile compounds identified in the flat peaches, using the peak areas of the internal standards as a benchmark. Further confirmation of the discriminant substances for the extent to which the compounds contribute to the aroma of the fruits was performed by calculating the OAVs of discriminant substances in flat peach based on the OT of volatile compounds in water. The OT referenced in this study was derived from the literature on previous studies [44,45,46,47].

### 2.9. Date Analysis

All results in this experiment were completed following three replicates of the assay. All histograms, visual graphs of correlation coefficients, and Venn diagrams in this article were created with Origin 2022 (Northampton, MA, USA). The distribution patterns of different fruit aromas were analyzed by drawing a PCA plot using Smica 14.1 (Umetrics, Sweden), and substances with OAVs greater than 1 were screened as characteristic aroma substances of the flat peach. The visualization of OAVs was drawn using TBtools v1.068, and the chemical structures of the screened characteristic aroma substances were plotted using Kingdraw 3.0 (Qingdao, China). All results measured in this test were analyzed by means of one-way analysis of variance (ANOVA) with the use of SPSS 19.0 (IBM, Chicago, IL, USA). Significant variations between samples are indicated when *p* < 0.05.

## 3. Results

### 3.1. The Assessment of Taste of Flat Peaches from Xinjiang

#### 3.1.1. The SSC and TA of Flat Peaches from Xinjiang

**Figure 2:**
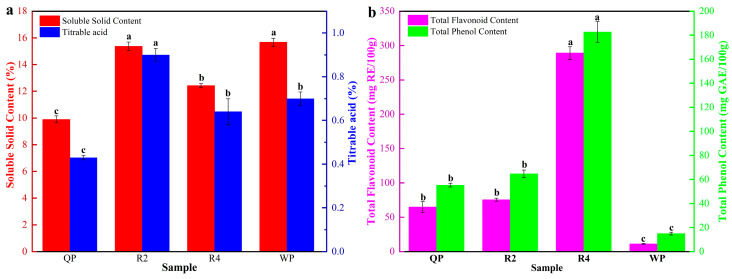
The soluble solid content, titratable acidity (**a**), total flavonoid content, and total phenol content (**b**) of flat peaches from Xinjiang. Significant variations at a level of *p* ≤ 0.05 (Duncan’s test) are shown by different letters for each sample.

It has been properly recognized that the SSC and TA of fruits are vital factors affecting their taste and, additionally, are important determinants of customer acceptability [48]. In this study, the SSC and TA of Qingpan (QP), Ruipan 2 (R2), Ruipan 4 (R4), and Wanpan (WP) were investigated. As is shown in Figure 2a, the SSC of the flat peaches ranged from 9.90% to 15.67%. WP showed the highest SSC (15.67%), while QP had the lowest SSC (9.90%). The TA of the flat peaches ranged from 0.43% to 0.90%. QP (0.43%) had the lowest TA and R2 (0.90%) had the highest TA. Of note, the SSC of the flat peaches ranged from 10% to 15% for all flat peaches except QP, and the TA of all tested flat peaches was less than 0.70%, except for R2. It has been documented that fruit in this range of SSC and TA are of high quality [49,50,51]. The results showed that the flat peaches from Xinjiang generally had a higher quality, broadly recognized by the industry. The SSC of nectarines was found to range from 9.5% to 14.4% and TA from 0.63 to 0.93% [52], and the “Xinchuanzhongdao” peach cultivar’s SSC ranged from 6.4% to 0.93% [53]. It is easy to observe the differences in SSC and TA as a result of the different cultivars. The SSC/TA ratio is also considered to be an important parameter to evaluate the taste equilibrium of fruits [54,55]. The SSC/TA ratio of the flat peaches was 17.04 to 22.98 (Supplementary Table S2). QP (22.98) and WP (22.36) had higher SSC/TA ratios, while R4 (19.46) and R2 (17.04) had slightly lower SSC/TA ratios. In general terms, this meant that the flat peaches had an excellent taste with high sugar and low acidity.

#### 3.1.2. The Composition and Content of Soluble Sugars and Organic Acids of Flat Peaches from Xinjiang

**Figure 3:**
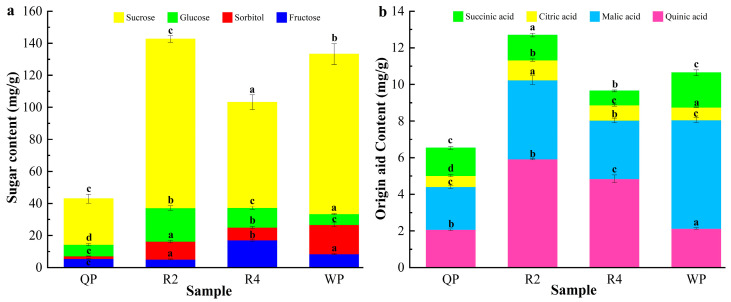
Soluble sugars (**a**) and organic acids (**b**) in flat peaches from Xinjiang (mg/g FW). Significant variations at a level of *p* ≤ 0.05 (Duncan’s test) are shown by different letters for each sample.

The composition and content of soluble sugars play a key role in consumer satisfaction and even determine the consumption of the best fruit [56]. In this study, the sucrose, fructose, glucose, and sorbitol contents of flat peaches (Qingpan, QP; Ruipan 2, R2; Ruipan 4, R4; Wanpan, WP) were determined. The results showed that sucrose, glucose, fructose, and sorbose accounted for 6.79~24.99%, 1.60~4.95%, 1.22~4.05%, and 0.39~4.34% of the total sugars, respectively (Supplementary Figure S1a). The contents of soluble sugar in flat peaches from Xinjiang were within 28.66 to 105.47 mg/g FW, 6.76 to 20.91 mg/g FW, 5.16 to 17.10 mg/g FW, and 1.66 to 18.31 mg/g FW, respectively (Figure 3a). The total sugar content ranged from 43.01 to 142.64 mg/g FW. R2 had the highest total sugar content, but QP had the lowest. Except for QP, the total sugar content of all three flat peaches exceeded 100 mg/g FW. The results were in agreement with earlier studies [57,58].

Sucrose, fructose, glucose, and sorbitol are the major sugar components of peaches [59]. In this study, sucrose and glucose were the major sugars in flat peaches, accounting for more than 80% of the total sugars, which was consistent with previous studies [60]. It was easy to see that sucrose was the main source of sweetness in the flat peaches, and glucose was the second most important source. The presence of sugars gives the fruit its sweetness, and the intensity of the sweetness is determined by the amount of total sugar and the proportion of sugar components [61]. The ratio of fructose to sorbose was found to be approximately 1:1 in the current study, and the glucose content was higher than the fructose content. Based on a previous study, the fructose sweetness scale was higher than that of glucose and sucrose [62], so it could be inferred that fructose is the largest contributor to the sweetness of flat peaches. Of note, there was some evidence to suggest that the fructose content was higher than the glucose content in peaches [63]. This discrepancy could be attributed to different seasonal changes, climate, irrigation, or crop loads.

According to previous reports, the content and composition of organic acids affected the acidity of fruits [64]. Four main organic acids, including quinic acid, malic acid, citric acid, and succinic acid, were determined from the flat peaches (Figure 3b). The results showed that the content of malic acid was highest, and the range was from 2.34 mg/g FW to 5.92 mg/g FW (5.93~14.96%) (Supplementary Figure S1b). Quinic acid (5.25% to 15.01%), which ranged from 2.07 mg/g FW to 5.93 mg/g FW, was the second most abundant organic acid after malic acid in the flat peaches. Succinic acid and citric acid made up a relatively small proportion of the organic acids in flat peaches, accounting for 14.08% and 8.14% of the total organic acid content, respectively. Among them, the succinic acid content ranged from 0.78 mg/g FW to 1.89 mg/g FW (1.97~4.78%) and the citric acid content ranged from 0.61 mg/g FW to 1.09 mg/g FW (1.55~2.75%). The current study showed that the total organic acid content of flat peaches ranged from 6.54 mg/g FW to 12.70 mg/g FW, with R2 having the highest total organic acid content, while QP was the lowest.

Several studies have shown that malic acid and citric acid were the main organic acids in peaches [41,60]; this was in line with our results. These results would seem to suggest that the content of organic acids in peach fruits was a discussable issue in identifying the style of peach. The results of this study showed that malic acid was the main source of sourness in the flat peaches, comprising 39.86% of the total organic acid content. The ratio of malic acid, quinic acid, citric acid, and succinic acid was approximately 2:2:1:1, which might be one of the reasons for the characteristic acidity of the taste of flat peaches. Similar to the effect of sugar on the sweetness of flat peaches, differences in the acidity of flat peaches were influenced not only by the absolute content of organic acids but also by the proportion of organic acids. In conclusion, the distribution and proportion of the content of sugars and organic acid compounds seemed to play a crucial role in influencing the sweetness and acidity of the flat peaches.

### 3.2. Assessment of Phenolic Compounds and Antioxidant Capacity of Flat Peaches from Xinjiang

#### 3.2.1. Analysis of Phenolic Compounds, TFC, and TPC in Flat Peaches from Xinjiang

Numerous studies have shown that phenolic compounds, as secondary metabolites of plants, are major contributors to antioxidant capacity [65]. Nine phenolic compounds have been identified in flat peaches (Qingpan, QP; Ruipan 2, R2; Ruipan 4, R4; Wanpan, WP) with HPLC (Table 1). Chlorogenic acid (0.22~32.29%) was the most abundant phenolic compound of the flat peaches from Xinjiang, with its content ranging from 1.58 to 29.49 mg/100 g FW (Supplementary Figure S2). Among them, R4 (29.49 mg/100 g FW) had the highest content of chlorogenic acid, about 20 times more than the other flat peaches. Proanthocyanidin B1 (0.82~19.92%) was the second most abundant phenolic compound of the flat peaches from Xinjiang, with concentrations ranging from 0.75 to 18.18 mg/100 g FW. In addition to the above, neochlorogenic acid and catechin were abundant in the flat peaches, ranging from 0.17 to 7.36 mg/100 g FW and 0.40 to 13.11 mg/100 g FW, accounting for 0.19~8.06% and 0.44~14.36% of the total phenolic compounds, respectively. The lower content of catechins compared to chlorogenic acid and neochlorogenic acid may be due to the fact that catechins are more easily oxidized, resulting in their reduced content [66]. The amounts of the remaining five phenolic compounds identified were relatively small in the tested flat peaches. Earlier studies have pointed out that the most important acid in peaches is chlorogenic acid, which has a high antioxidant capacity [67].

As one kind of polyphenolic compound, flavonoids are also of great significance to human health. As shown in Figure 2b, TFC ranged from 11.30 to 289.02 mg RE/100 g and TPC ranged from 15.07 to 182.80 mg GAE/100 g in the flat peaches, which was also consistent with previous research findings [68]. R4, with the highest TFC (289.02 mg RE/100 g), was 20 times higher than WP, with the lowest content (11.30 mg RE/100 g). Additionally, R4 had the highest TFC and TPC, hence, it could be considered that its medicinal value was higher than the others, and its components could be extracted and processed into health products later.

#### 3.2.2. Antioxidant Activity In Vitro in Flat Peaches from Xinjiang

Antioxidant polyphenolic compounds provide hydrogen ions, electrons, and metal chelation, which are beneficial to human health [31,69]. The ABTS^+^ radical scavenging capacity and DPPH radical scavenging rate, CUPRAC, and FRAP reducing capacity were determined to characterize the antioxidant activity of phenolic compounds in flat peaches (Qingpan, QP; Ruipan 2, R2; Ruipan 4, R4; Wanpan, WP) from Xinjiang, shown in Table 2. The ABTS^+^ radical scavenging capacity and DPPH radical scavenging rate of the flat peaches ranged from 165.40 to 324.38 μmol TEs/100 g and 207.93 to 799.16 μmol TEs/100 g, respectively. R4 had the highest ABTS^+^ radical scavenging capacity and DPPH radical scavenging rate, but WP was the lowest. This may also be due to the high content of hydrogen donor molecules in flat peaches, which could reduce the production of free radicals, thus reflecting higher free radical scavenging activity. In addition, FRAP and CUPRAC reducing capacities are other important indicators reflecting the potential antioxidant activity in flat peaches. In the experiment, the CUPRAC reducing capacity of the flat peaches was between 44.69 and 1145.90 μmol TEs/100 g, and the FRAP reducing capacity of the flat peaches ranged from 57.56 to 1252.00 μmol TEs/100 g. More importantly, R4 exhibited superior antioxidant capacity; two to three times more than the other three flat peaches.

Early research reported that antioxidant capacity assay in vitro could reveal the main functional characteristics of fruit tissues [70]. In the present study, it was revealed that R4 demonstrated a strong antioxidant capacity in vitro. The antioxidant effect of peaches has been demonstrated in previous experiments [67,71]. Based on the results of this study, flat peaches have significant potential as an antioxidant ingredient in animal feed or human health food.

#### 3.2.3. Correlation Analysis of Phenolic Compounds and Antioxidant Capacity of Flat Peaches from Xinjiang

**Figure 4:**
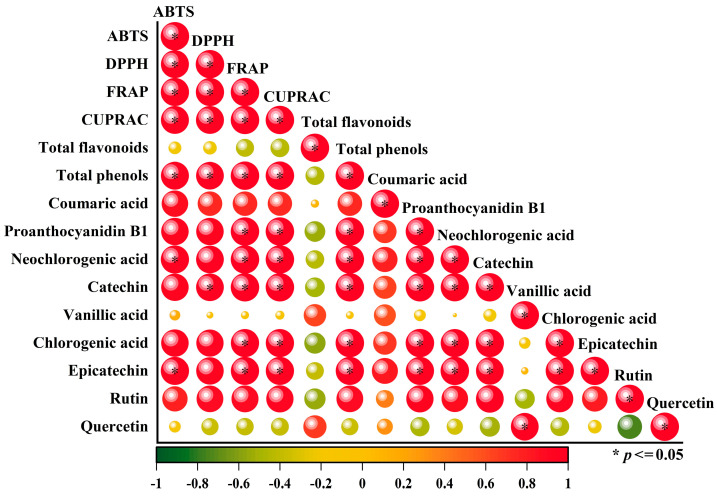
The Pearson rank correlation matrix between the phenolic compounds and antioxidant activity of flat peaches from Xinjiang. High and low correlations are shown by the large and small circles, respectively. When the scale bar is red and the value is 1, this indicates a perfectly positive correlation; when the scale bar is green and the value is −1, this indicates a perfectly negative correlation. Significant correlations with a level of *p* < 0.05 are shown by *.

Antioxidant capacity is generally the basic ability of phenolic compounds to prevent or treat diseases [72]. To investigate the relationship between phenolic compounds and antioxidant activity in flat peaches, with polyphenol and flavonoid content as independent variables and antioxidant activity as the dependent variable, the following results were obtained by multi-dimensional data analysis using O2PLS models combined with correlation coefficients. When the VIP value was more than 1, this proved that the phenolic compounds were more correlated with the antioxidant capacity. Proanthocyanidin B1 (D), chlorogenic acid (H), catechin (F), neochlorogenic acid (E), epicatechin (I), rutin (J), and total phenols (B) correlated more strongly with antioxidant capacity (Supplementary Figure S3). In Figure 4, the correlation coefficient matrix reveals that epicatechin, rutin, catechin, proanthocyanidin B1, chlorogenic acid, neochlorogenic acid, and total phenols were positively correlated with ABTS^+^, DPPH, CUPRAC, and FRAP. Total flavonoids and quercetin were negatively correlated in the opposite direction. Interestingly, vanillic acid was positively correlated with ABTS^+^, while it was negatively correlated with DPPH, FRAP, and CUPRAC, which also suggests that vanillic acid was one of the main sources of ABTS^+^ radical scavenging capacity. Previous studies have described a significant positive correlation between phenolic compounds and the antioxidant capacity of peaches [73].

### 3.3. Construction of Aroma Fingerprinting of Flat Peaches from Xinjiang

#### 3.3.1. The Composition and Content of Volatile Compounds of Flat Peaches from Xinjiang

**Figure 5:**
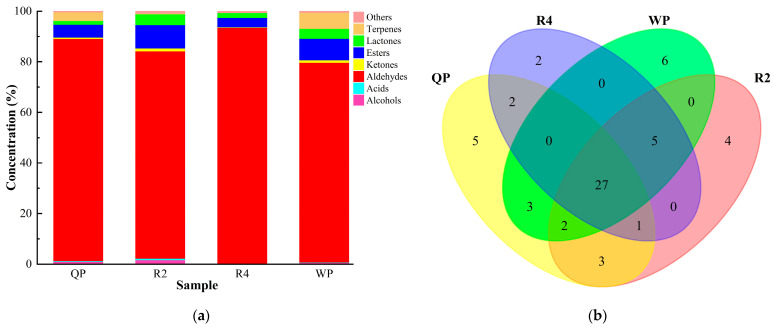
Classification of volatile aroma compounds of flat peaches from Xinjiang. (**a**) The relative composition of volatile aroma substances of flat peaches; (**b**) the Venn diagram of the volatile compounds of flat peaches.

Volatile aroma substances are the primary or secondary metabolites of fruits, which can reflect their aroma pattern and affect their taste and flavor expression [74]. HS-SPME-GC-MS was used to analyze the volatile aroma compounds of samples of flat peaches (Qingpan, QP; Ruipan 2, R2; Ruipan 4 R4; Wanpan, WP). A total of 60 volatile aroma compounds were identified from the flat peaches, including four alcohols, two acids, eighteen aldehydes, five ketones, nine terpenes, sixteen esters, seven lactones, and two terpenes (Supplementary Table S4). As shown in Figure 5a, the first-ranked volatile compounds in the tested flat peaches were aldehydes (10.09~31.98%), followed by esters (1.15~2.16%) and terpenes (0.04~1.63%). It was noteworthy that no terpenoids were detected in R4. Lactones, alcohols, and ketones accounted for 0.40–1.00%, 0.04–0.28%, and 0.07–0.24% of the total volatile compounds in the flat peaches, respectively. Acids only accounted for 0.03–0.07% of the total volatile compounds of flat peaches. With regards to our study, benzaldehyde was the most abundant volatile compound in the flat peaches and comprised 4.74~27.28% of the total volatile aroma compounds and approximately 5.47~31.46% of the total aldehydes. 2-Hexenal (4.23~9.35%), which comprised approximately 3.67~8.11% of the total volatile compounds, was the second-most abundant aldehyde. 3-hexenyl acetate (1.51~18.69%) and phenethyl acetate (3.52~7.97%) were important components of esters in flat peaches (Supplementary Table S4). In addition, we found that a total of 27 volatile aroma substances were evaluated in all four tested flat peaches (Figure 5b). In summary, aldehydes (especially benzaldehyde and 2-hexenal) and esters were the main aroma compounds of flat peaches from Xinjiang.

#### 3.3.2. PCA of Volatile Compounds of Flat Peaches from Xinjiang

**Figure 6:**
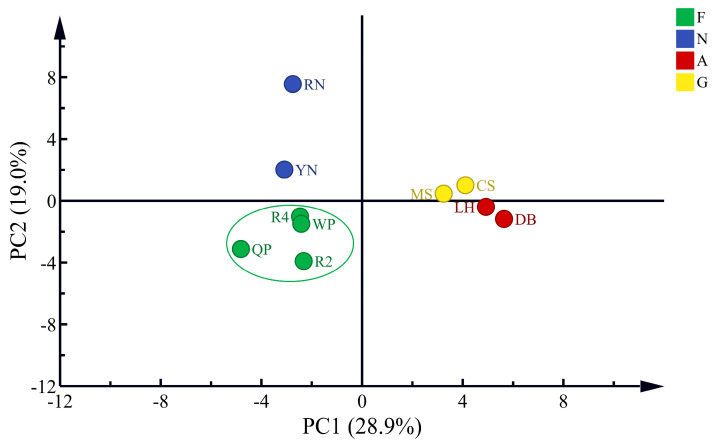
PCA of volatile compounds in dominant fruits from Xinjiang. F: flat peach (*P. persica* L. *Batsch. var. compressa Bean*), including Qingpan (QP), Ruipan 2 (R2), Ruipan 4 (R4), and Wanpan (WP); N: nectarine (*Prunus nucipersica* L.), including yellow nectarine (YN) and red nectarine (RN); A: apricot (*Prunus armeniaca* L.), including Dabaiyou (DBY) and Luopuhongdaike (LH); G: grape (*Vitis vinifera* L.), including Marselan (MS) and Cabernet Sauvignon (CS). Data from the same laboratory, adapted from Huimin Wu et al. (2022) [76], Cai Zhao et al. (2022) [77], and Yuanyuan Miao et al. (2022) [78].

In the experiments, to investigate the intergroup differences in the volatile compounds of peaches, data on the volatile compounds of nectarines (YN and RN), apricots (DB and LH), and grapes (CS and MS) were introduced, and 151 volatile substances were analyzed with PCA (Figure 6). It is not difficult to see that the four flat peaches and their 151 volatile compounds were clustered to form the corresponding groupings. Two major components explained 48.4% of the total variance, with PC1 accounting for 28.9% of the total variance and PC2 accounting for 19.0% of the total variance. Among them, the flat peaches (QP, R2, R4, and WP) were classified as group F, probably due to the similarities between them. Similarly, YN and RN were classified as group N, DB and LH were classified as group A, and CS and MS were classified as group G. That flat peaches were mainly concentrated in the lower half of the left negative half-axis stemmed from the similar aroma characteristics. Flat peaches and nectarines are both peach varieties and thus were concentrated in the left half-axis. This also revealed the presence of similar aromatic substances in flat peaches and nectarines. The results show a distinctly similar aroma between the flat peaches. The loading diagram provided the distribution of 151 volatile compounds of four species (Supplementary Figure S4). Flat peaches were characterized by 2-phenylethyl caproate, benzaldehyde, 2-hexenal, 2,4-heptadienal, γ-octalactone, and 2-hexenyl acetate, which had a positive correlation with flat peaches. It was verified earlier that the volatile compounds in peaches vary from species to species [75].

#### 3.3.3. Screening of Common Aromatic Substances in Flat Peaches from Xinjiang

**Figure 7:**
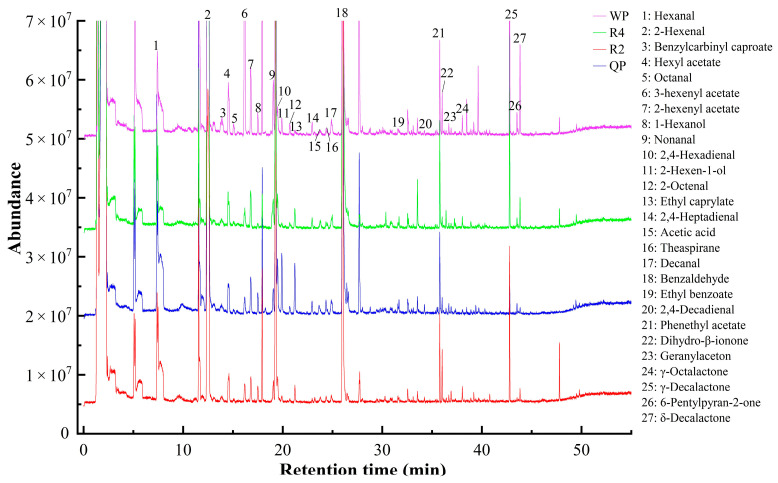
Overlay of the common ion chromatograms by HS-SPME-GC-MS analysis of flat peaches from Xinjiang.

Although peach varieties have a large number of volatile compounds, some of them have similar aroma characteristics. 2-Hexenal, benzaldehyde, γ-octalactone, phenethyl acetate, and 6-pentyl-2H-pyran-2-one are commonly found in peach varieties (Supplementary Figure S5). To investigate the aroma characteristic of flat peaches (Qingpan, QP; Ruipan 2, R2; Ruipan 4 R4; Wanpan, WP) from Xinjiang, 27 volatile substances with high content were screened as common aromatic substances of flat peaches from Xinjiang (Figure 7). The results showed that the flat peaches possessed similar volatile aroma compounds. 2-Hexenal, decanal, benzaldehyde, γ-decalactone, δ-decalactone, and 3-hexenyl acetate were common compounds in the flat peaches, and these compounds had a high concentration. It was confirmed earlier that the concentration of volatile compounds was negatively correlated with the degree of aroma contribution, and higher volatile concentration did not mean a greater impact on the aroma. Thus, the contribution of volatile aroma compounds to odor characteristics was examined by means of calculating the odor activity value (OAV), and the contribution of volatile compounds to the aroma was examined only when OAV > 1 [79].

**Figure 8:**
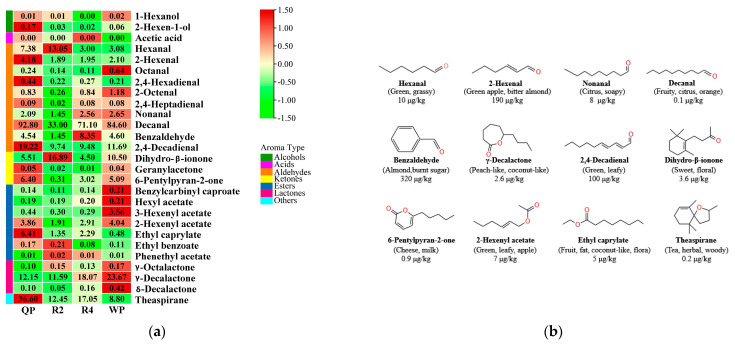
The heatmap analysis of the OAVs of selected main volatile compounds found in flat peaches from Xinjiang. (**a**) Heatmap visualization of the analysis of OAVs. For the same compound, red (1.5) represents the maximum OAV and green (−1.5) represents the minimum OAV. (**b**) Information on the key aroma characteristic compounds. Twelve key aroma compounds in flat peaches. The odor thresholds (OT) of the compounds in water are reported in the literature.

Among the 27 volatile compounds in flat peaches, the OAV of 12 volatile aroma substances was more than 1, primarily hexanal, 2-hexenal, nonanal, decanal, benzaldehyde, 2,4-decadienal, dihydro-β-ionone, 6-pentylpyran-2-one, ethyl caprylate,2-hexenyl acetate, γ-decalactone, and theaspirane (Figure 8a). It is easy to see that decanal (OAV 33.00–92.80), theaspirane (OAV 8.80–36.60), γ-decalactone (OAV 11.59–23.67), 2,4-decadienal (OAV 9.48–19.22), dihydro-β-ionone (OAV 4.50–16.89), and hexanal (OAV 3.00–13.05) had the highest levels of OAV. Conversely, the OAVs of nonanal (OAV 1.45–2.65), 2-hexenal (OAV 1.89–4.18), and 2-hexenyl acetate (OAV 1.91–4.04) were lower. Furthermore, as shown in Figure 8b, 2,4-decadienal (0.73–1.48 μg/kg) and 6-pentylpyran-2-one (0.28–5.76 μg/kg) were present only in trace amounts but were also considered as strong aroma contributors to the flat peaches from Xinjiang due to their extremely low odor threshold (0.077 μg/kg, 0.90 μg/kg). The odor characteristics of the flat peaches might be due to the presence of these 12 volatile compounds. Several studies have shown that C_6_ compounds of aldehydes (including hexenal and 2-hexenal), lactones (γ-decalactone and δ-dodecalactone), and esters (especially hexyl acetate) play a key role in the typical aroma of flat peaches [80]. Hexanal and 2-hexenal render “fruity” and “grassy” aromas for flat peaches, γ-decalactone is an important source of “peach-like” aroma, and benzaldehyde and lactones in synergy to give “almond” and “burnt sugar” aromas to flat peaches [81,82]. In the report by Xi Wanpeng et al. (2017) [83], it was also noted that volatile compounds such as linalool, methyl acetate, ethyl acetate, isobutyl acetate, hexanol, and benzene ethanol were closely related to the aroma of flat peaches.

#### 3.3.4. Identification of Characteristic Aroma Fingerprint of Flat Peaches from Xinjiang

**Figure 9:**
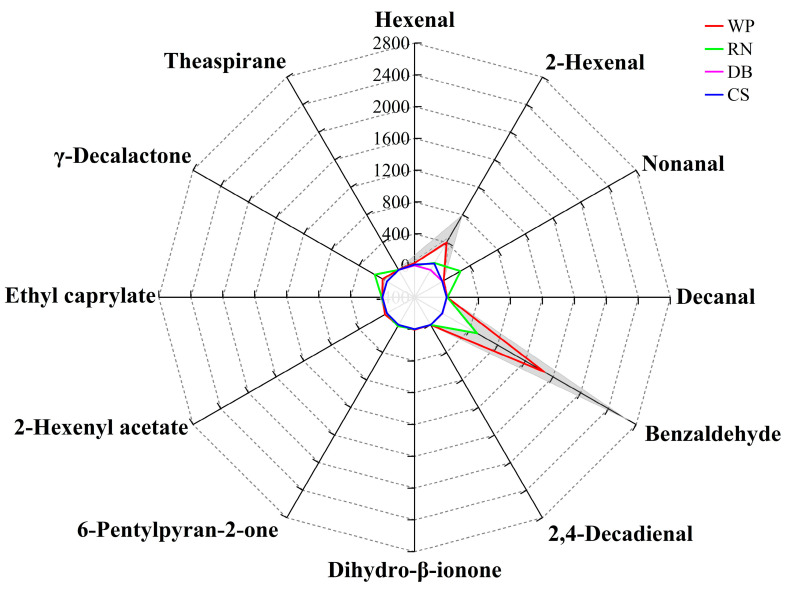
The aroma fingerprint of flat peaches from Xinjiang. The confidence interval and average values of 12 volatile compound concentrations in flat peaches, including Qingpan (QP), Wanpan (WP), Ruipan 2 (R2), and Ruipan 4 (R4). Yellow line is nectarine (*P. nucipersica* L.), yellow nectarine (YN); red line is apricot (*P. armeniaca* L.), Dabaiyou (DBY); blue line is grape (*V. vinifera* L.), Cabernet Sauvignon (CS); green line is R4 (flat peach).

To verify the aroma fingerprinting of flat peaches from Xinjiang, hexanal, 2-hexenal, nonanal, decanal, benzaldehyde, 2,4-decadienal, dihydro-β-ionone, 6-pentylpyran-2-one, 2-hexenyl acetate, ethyl caprylate, γ-decalactone, and theaspirane, which had OAVs greater than 1, constituted the aroma fingerprinting of flat peaches from Xinjiang (Figure 9). Twelve volatile key compounds from nectarines (RN), apricots (DB), and grapes (CS) were introduced and their concentrations were compared (Supplementary Table S4). The shaded area shows the confidence interval for the major aromas in the flat peaches from Xinjiang, and the solid black line shows the average value (Figure 9). The aroma fingerprinting of RN, mainly including benzaldehyde, 6-pentylpyran-2-one, and ethyl caprylate, was similar to the aroma fingerprinting of the flat peaches, to some extent. What is interesting about the data in this figure is that 2,4-decadienal, 2-hexenyl acetate, and theaspirane were only found in flat peaches, and these concentrations were consistent with the confidence intervals of the four flat peaches, so three compounds could be aroma characteristic substances identifying flat peaches from Xinjiang. Taken together, these results suggest that flat peaches have a unique aroma and provide a theoretical basis for the rapid identification of flat peaches with other peach varieties and quality control of the flavor of flat peach products.

The odor characteristics of flat peaches are summarized in Supplementary Figure S4. The results showed that the aroma characteristics of flat peaches were basically the same; the odor characteristics mainly showed “fruit”, “floral”, “peach-like”, and “coconut-like” aromas, with the “fruit” aroma being more prominent. It should be noted that QP had significantly higher “tea” and “fatty” aromas in the model. Among the flat peaches, QP had the highest OAV, followed by WP and R4, and the lowest was R2.

## 4. Conclusions

In this study, the physicochemical characteristics, phenolic compounds, antioxidant activity, and volatile compounds of flat peaches were investigated in terms of taste, antioxidant capacity, and odor characteristics, constructing an aroma fingerprinting of flat peaches from Xinjiang. These results suggest that flat peaches have the trait of high sugar–low acid; the total sugar content of the flat peaches ranged from 43.01 mg/g FW to 142.64 mg/g FW, and sucrose (63.86~73.86%) was the main sugar. Meanwhile, the total organic acid content of the flat peaches ranged from 6.54 mg/g FW to 12.70 mg/g FW, and the organic acids were dominated by malic acid (5.93~14.96%) and quinic acid (5.25~15.01%). The ratio of fructose to sorbose was found to be approximately 1:1, and the ratio of malic acid, quinic acid, citric acid, and succinic acid was approximately 2:2:1:1, which might be one of the reasons for the special taste of flat peach. Flat peaches possess a high level of phenolic compounds, which endue the flat peaches with high antioxidant activity. The ABTS^+^ and DPPH radical scavenging capacity in the flat peaches ranged from 165.40 to 324.38 μmol TEs/100 g and 207.93 to 799.16 μmol TEs/100 g, respectively. The CUPRAC reducing capacity of the flat peaches ranged from 44.69 to 1145.90 μmol TEs/100 g and the FRAP reducing capacity of the flat peaches ranged from 57.56 to 1252.00 μmol TEs/100 g. Chlorogenic acid (0.22~32.29%), epicatechin, rutin, catechin, proanthocyanidin B1, and neochlorogenic acid were positively related to the antioxidant activity of the flat peaches.

In addition, the aroma characteristics of the flat peaches were systematically characterized, and the volatile compounds of the flat peaches were found to consist of four alcohols, two acids, eighteen aldehydes, five ketones, nine terpenes, sixteen esters, seven lactones, and two terpenes. Among them, aldehydes (especially benzaldehyde and 2-hexenal) and esters were the main volatile components. A total of 20 common volatile compounds between flat peaches and nectarines were found in the study, indicating that flat peaches and nectarines have some similarities. However, the flat peaches had unique odor characteristics. A total of 27 common volatile substances were identified in the flat peaches, of which hexanal, 2-hexenal, nonanal, decanal, benzaldehyde, 2,4-decadienal, dihydro-β-ionone, 6-pentylpyran-2-one, 2-hexenyl acetate, ethyl caprylate, γ-decalactone, and theaspirane constituted the aroma fingerprinting of the flat peaches from Xinjiang. In particular, 2,4-decadienal, theaspirane, and 2-hexenyl acetate could be used as tracers, which can help to quickly distinguish flat peaches from other peaches by aroma criteria. Additionally, the odor characteristics of the flat peaches from Xinjiang were distinctly “peach-like”, “fruit”, and “coconut-like”. This study was only a preliminary study on the aroma characteristics of flat peaches from Xinjiang. In the future, the formation mechanism of flavor in flat peaches can be studied in depth using techniques such as metabolomics to lay the theoretical foundation for the development of the industry of flat peaches in Xinjiang.

## Figures and Tables

**Table 1:** The content and composition of phenolic compounds in flat peaches from Xinjiang (mg/100 g FW).

Variety	QP	R2	R4	WP
Coumaric acid	0.19 ± 0.01 b	0.39 ± 0.03 a	0.43 ± 0.03 a	0.13 ± 0.01 c
Proanthocyanidin B1	2.77 ± 0.22 b	2.05 ± 0.16 c	18.19 ± 0.54 a	0.75 ± 0.05 d
Neochlorogenic acid	1.12 ± 0.06 c	1.81 ± 0.07 b	7.36 ± 0.15 a	0.17 ± 0.02 d
Catechin	3.14 ± 0.05 b	1.46 ± 0.04 c	13.11 ± 0.48 a	0.40 ± 0.02 d
Vanillic acid	0.08 ± 0.01 c	0.49 ± 0.02 a	0.14 ± 0.01 b	0.08 ± 0.01 c
Chlorogenic acid	1.58 ± 0.02 b	1.82 ± 0.16 b	29.49 ± 1.79 a	0.20 ± 0.02 b
Epicatechin	0.13 ± 0.01 c	0.44 ± 0.04 b	1.49 ± 0.11 a	n. d.
Rutin	0.26 ± 0.01 b	0.12 ± 0.02 d	0.43 ± 0.02 a	0.17 ± 0.02 c
Quercetin	0.14 ± 0.01 c	0.49 ± 0.01 a	0.11 ± 0.01 d	0.19 ± 0.03 b

“n. d.” indicates that the substance was not detected in the corresponding sample. Each value was the mean ± standard deviation. Significant variations with a level of *p* ≤ 0.05 (Duncan’s test) are shown by different letters for each sample.

**Table 2:** Antioxidant ability in vitro of flat peaches from Xinjiang (μmol TEs/100 g FW).

Variety	Radical Scavenging Capacity		Reducing Capacity	
	ABTS ^+^	DPPH	CUPRAC	FRAP
QP	324.38 ± 14.31 c	464.54 ± 12.87 b	278.50 ± 13.91 b	297.56 ± 3.85 b
R2	404.41 ± 24.33 b	398.04 ± 17.27 c	274.93 ± 8.78 b	279.78 ± 8.39 b
R4	717.61 ± 3.29 a	799.16 ± 5.57 a	1145.90 ± 26.32 a	1252.00 ± 67.66 a
WP	165.40 ± 13.71 d	207.93 ± 12.81 b	44.69 ± 3.52 c	57.56 ± 5.09 c

Each value was the mean ± standard deviation. Significant variations with a level of *p* ≤ 0.05 (Duncan’s test) are shown by different letters for each sample. ^+^ denoted as cation.

## Data Availability

Data are contained within the article.

## Supplementary Materials

The following supporting information can be downloaded at: https://www.mdpi.com/article/10.3390/foods12132554/s1, Table S1: Fruit fresh weight, moisture content, diameter, and firmness of flat peaches from Xinjiang at commercially mature stages; Table S2: Fundamental physicochemical data of flat peaches from Xinjiang; Table S3: Concentration of volatile compounds of flat peaches from Xinjiang (μg/kg); Table S4: Tentative identifications of volatile compounds of flat peaches from Xinjiang (μg/kg); Table S5: Concentration of the main aroma compounds of flat peaches, nectarines, apricots, and grapes (μg/kg); Figure S1: The concentration of soluble sugars and organic acids in flat peaches from Xinjiang; Figure S2: The concentration of phenolic compounds in flat peaches from Xinjiang; Figure S3: Importance of variables between phenolic compounds and antioxidant activity of flat peaches from Xinjiang; Figure S4: The loading diagram of PCA of volatile compounds of flat peaches from Xinjiang; Figure S5: Overlay of the common ion chromatograms in the HS-SPME-GC-MS analysis of flat peaches and nectarines; Figure S6: Odor characteristics of the main aroma substances of flat peaches from Xinjiang.
