# Supplementary material for: Flavor Characterization of Native Xinjiang Flat Peaches Based on Constructing Aroma Fingerprinting and Stoichiometry Analysis
Source: Foods. 2023 Jun 29;12(13):2554. doi: 10.3390/foods12132554 (PMC10341215; doi:10.3390/foods12132554)
Supplement: Supplementary file 1 [file foods-12-02554-s001.zip › foods-2466168-supplementary.pdf]

Supplementary file for:

Flavor characterization of native Xinjiang flat peaches based on constructing aroma fingerprinting and stoichiometry analysis

Chunyan Li, Youyou Xu, Huimin Wu, Ruiui Zhao, Xinwei Wang, FangFang Wang, Qingquan Fu, Tiantian Tang, Xuewie Shi, Bin Wang *

Food college, Shihezi University, Shihezi 832000, Xinjiang, People' s Republic of China (PRC).

* Correspondence: B. W.: binwang0228@shzu.edu.cn Tel: 86-0993-2058093

Contents

Supplementary Tables 3
Table S1. Fruit fresh weight, moisture content, diameter and firmness of flat peach in Xinjiang at commercially mature stages. 3
Table S2. Fundamental physicochemical data of flat peach in Xinjiang. 3
Table S3. Concentration of volatile compounds of flat peach in Xinjiang (μg/kg). 3
Table S4. Tentative identifications of volatile compounds of flat peach in Xinjiang (μg/kg). 4
Table S5. Concentration of main aroma compounds of flat peaches, nectarine, apricot and grape (μg/kg). 7
Supplementary Figures 8
Figure S1. The concentration of soluble sugars and organic acids of flat peach in Xinjiang. 8
Figure S2. The concentration of phenolic compounds of flat peach in Xinjiang. 8
Figure S3. Importance of variables between phenolic compounds and antioxidant activity of flat peach in Xinjiang. 9
Figure S4. The loading diagram of PCA of volatile compounds of flat peach in Xinjiang. 10
Figure S5. Overlay of the common ion chromatograms in the HS-SPME-GC-MS analysis of flat peaches and nectarines. 11
Figure S6. Odor characteristics of main aroma substances of flat peach in Xinjiang. 12

Supplementary Tables

Table S1. Fruit fresh weight, moisture content, diameter and firmness of flat peach in Xinjiang at commercially mature stages.

Variety	Fresh weight (g)	Moisture content (%)	Transverse diameter (mm)	Longitudinal diameter (mm)	Firmness (N)
QP	126.99±8.03b	85.44±0.41b	77.27±3.68b	71.79±3.04b	12.72±1.03a
R2	105.32±5.08c	82.78±0.13c	68.47±1.01c	66.63±1.22c	9.05±0.64c
R4	105.91±5.82c	86.59±0.36a	70.96±2.39c	66.97±3.41c	12.43±0.32a
WP	193.08±10.16a	85.51±0.37b	86.17±2.59a	74.64±2.72a	11.96±1.04b

Each value was the mean ± standard deviation. Significant variations are shown with different letters for each sample at a level of $p \leq 0.05$.

Table S2. Fundamental physicochemical data of flat peach in Xinjiang.

Variety	Soluble Solid Content (%)	Titrable Acidity (%)	Total Flavonoid Content mg RE/100g	Total Phenol Content mg GAE/100g	SSC/TA Ratio
QP	9.90±0.26c	0.43±0.01c	64.98±7.90b	55.36±1.55b	22.98±0.66a
R2	15.37±0.31a	0.90±0.03a	75.51±2.19b	64.71±3.11b	17.04±0.64b
R4	12.43±0.15b	0.64±0.06b	289.02±9.27a	182.80±8.79a	19.46±2.00b
WP	15.67±0.31a	0.70±0.03b	11.30±0.80c	15.07±1.14c	22.36±0.82a

Each value was the mean ± standard deviation. Significant variations are shown with different letters for each sample at a level of $p \leq 0.05$.

Table S3. Concentration of volatile compounds of flat peach in Xinjiang (μg/kg).

Variety	Alcohols	Acids	Aldehydes	Ketones	Esters	Lactones	Terpenes	Others
QP	27.85	6.87	2412.30	13.65	142.00	39.36	95.11	8.84
R2	19.96	6.82	988.50	13.18	112.27	50.55	4.30	10.00
R4	3.85	6.34	3132.39	6.70	125.28	66.95	n. d.	19.75
WP	15.66	3.13	1960.41	23.97	211.51	97.74	159.30	11.78

"n. d." indicates that the substance was not detected in the corresponding sample. Each value was the mean ± standard deviation. Significant variations are shown with different letters for each sample at a level of $p \leq 0.05$.

Table S4. Tentative identifications of volatile compounds of flat peach in Xinjiang (μg/kg).

Tentative I.D.	RI	QP	R2	R4	WP
**Alcohols**					
1-Hexanol	1340	7.29±0.15b	6.49±0.14c	1.96±0.02d	7.91±0.22a
2-Hexen-1-ol	1398	16.61±0.43a	2.66±0.07c	1.89±0.03c	5.58±0.15b
1-Octanol	1558	2.82±0.16a	9.07±0.29a	n. d.	n. d.
Dihydro-β-ionol	1976	1.13±0.02c	1.74±0.04b	n. d.	2.17±0.06a
**Acids**					
Acetic acid	1461	5.50±0.12b	4.69±0.11c	6.34±0.13a	3.13±0.08d
Pentanoic acid	1734	1.37±0.04b	2.13±0.07a	n. d.	n. d.
**Aldehydes**					
Hexanal	1081	74.83±1.78b	130.55±3.58a	29.95±0.90c	30.85±1.00c
2-Methylpent-4-enal	1141	0.74±0.02b	n. d.	n. d.	n. d.
3-Methyl hexanal	1156	n. d.	n. d.	n. d.	2.21±0.06a
2-Hexenal	1213	794.00±13.92a	359.12±8.92c	371.43±9.12c	399.19±10.09b
Octanal	1298	1.65±0.02b	0.95±0.01c	0.75±0.01d	4.44±0.14a
Nonanal	1396	16.75±0.43c	11.59±0.24d	20.51±0.81b	21.16±0.89a
2,4-Hexadienal	1411	26.57±0.70a	13.32±0.33c	16.46±0.51b	12.51±0.32d
2-Octenal	1427	2.50±0.05b	0.77±0.01c	2.51±0.05b	3.53±0.10a
2,4-Heptadienal	1469	5.18±0.17a	1.01±0.04c	4.23±0.14b	4.32±0.12b
Furfural	1472	n. d.	1.45±0.03a	n. d.	n. d.
Decanal	1510	9.28±0.49a	3.3±0.09d	7.11±0.19c	8.46±0.29b
Benzaldehyde	1530	1454.10±41.47b	464.47±18.64c	2672.29±50.69a	1472.84±41.38b
2-Nonenal	1548	24.50±0.61a	n. d.	n. d.	n. d.
2,6-Nonadienal	1715	1.14±0.05a	n. d.	n. d.	n. d.
Phenylacetaldehyde	1722	0.58±0.01b	n. d.	5.20±0.11a	n. d.
2,4-Decadienal	1766	1.48±0.06a	0.75±0.01c	0.73±0.01c	0.90±0.01b
2,5-Dimethylbenzaldehyde	1775	n. d.	n. d.	1.22±0.04a	n. d.
5-Hydroxymethylfurfural	2528	n. d.	1.22±0.04a	n. d.	n. d.
**Ketones**					
3-Octanone	1251	n. d.	n. d.	n. d.	3.31±0.08a
Dihydro-β-ionone	1825	3.86±0.08c	11.82±0.58a	3.15±0.10d	7.35±0.25b
Geranylacetone	1867	2.99±0.09a	1.08±0.04c	0.83±0.01d	2.34±0.03b
β-ionone	1954	1.04±0.04b	n. d.	n. d.	6.39±0.17a
6-Pentyl-2H-pyran-2-one	2175	5.76±0.11a	0.28±0.01d	2.72±0.05c	4.58±0.08b
**Esters**					
Benzylcarbinyl caproate	1822	5.87±0.19b	4.91±0.12c	5.85±0.15b	9.20±0.29a
Hexyl acetate	1269	21.86±0.64c	22.30±0.78bc	22.83±0.76b	24.35±0.88a
3-Hexenyl acetate	1309	13.51±0.43b	9.30±0.19b	8.90±0.10b	110.45±5.55a
Vinyl hexanoate	1334	n. d.	0.64±0.02a	n. d.	n. d.
2-Hexenyl acetate	1337	27.05±0.64b	13.35±0.21d	20.37±0.56c	28.29±0.66a
Heptyl Acetate	1377	2.63±0.07a	n. d.	n. d.	0.87±0.02b

Table S4 Continued

Methyl octylate	1397	n. d.	n. d.	5.76±0.12a	n. d.
Ethyl caprylate	1436	32.03±1.05a	6.76±0.16c	11.46±0.43b	2.41±0.05d
Methyl benzoate	1616	n. d.	n. d.	n. d.	0.63±0.02a
Methyl-4-decenoate	1620	3.75±0.10a	n. d.	3.67±0.09a	n. d.
Ethyl caprate	1630	n. d.	2.43±0.07a	0.88±0.02b	0.80±0.01b
3-Hexenylbutyrate	1647	n. d.	n. d.	n. d.	1.03±0.04a
Ethyl benzoate	1658	3.83±0.14b	4.65±0.18a	1.70±0.06d	2.47±0.07c
Ethyl-4-decenoate	1672	9.48±0.23a	0.83±0.01c	2.90±0.09b	n. d.
Hexyl octanoate	1800	1.16±0.04a	n. d.	n. d.	n. d.
Phenethyl acetate	1807	20.83±0.71d	47.10±1.01a	40.96±0.95b	31.01±0.82c
**Lactones**					
γ-Caprolactone	1709	n. d.	3.44±0.09c	4.09±0.14b	8.13±0.23a
γ-Heptalactone	1784	0.33±0.01a	n. d.	n. d.	n. d.
γ-Octalactone	1886	2.29±0.03d	3.67±0.10b	3.13±0.06c	4.08±0.07a
δ-Octalactone	1967	n. d.	n. d.	n. d.	0.28±0.01a
γ-Decalactone	2130	31.58±0.79c	30.13±0.76d	46.97±1.13b	61.53±1.43a
δ-Decalactone	2193	5.16±0.13c	2.49±0.10d	8.16±0.26b	21.57±0.84a
γ-Dodecalactone	2376	n. d.	10.82±0.49a	4.60±0.12b	2.15±0.08c
**Terpenes**					
β-Myrcene	1145	n. d.	n. d.	n. d.	2.73±0.10a
Linalool	1537	95.11±1.65b	4.3±0.15c	n. d.	156.57±6.06a
**Others**					
Paraxylene	1139	n. d.	1.06±0.03a	n. d.	n. d.
2-Pentylfuran	1228	n. d.	2.74±0.10b	1.43±0.05c	6.25±0.20a
Theaspirane	1523	7.32±0.25a	2.49±0.08c	3.41±0.09b	1.76±0.05d
Azulene	1746	n. d.	2.97±0.05b	14.91±0.6a	3.44±0.77b
2-Methylnaphthalene	1852	1.04±0.03a	0.74±0.01b	n. d.	n. d.
15-Crown-5	2431	0.48±0.01a	n. d.	n. d.	0.33±0.01b

"n. d." indicates that the substance was not detected in the corresponding sample. Each value was the mean ± standard deviation. Significant variations are shown with different letters for each sample at a level of $p \leq 0.05$.

Table S5. Concentration of main aroma compounds of flat peaches, nectarine, apricot and grape (μg/kg).

Compounds	RI	WP	RN	DB	CS	Average	Confidence interval
Hexanal	1081	30.85	3.98	2.37	13.32	66.30	66.30±47.78
2-Hexenal	1213	399.19	97.86	0	91.25	480.94	480.94±209.38
Nonanal	1396	21.16	263.90	3.25	0	17.50	17.50±4.39
Decanal	1510	8.46	11.15	1.98	0	7.04	7.04±2.65
Benzaldehyde	1530	1472.80	502.47	1.73	0	1515.93	1515.93±903.40
2,4-Decadienal	1766	0.90	0	0	0	0.97	0.97±0.35
Dihydro-β-ionone	1825	7.35	0.86	0	0	6.55	6.55±3.97
6-Pentylpyran-2-one	2175	4.58	17.99	0	0	3.34	3.44±22.39
2-Hexenyl acetate	1337	28.29	0	0	0	22.27	22.27±6.89
Ethyl caprylate	1630	2.41	10.57	0	3.78	13.17	13.17±13.11
γ-Decalactone	2130	61.53	176.52	0	0	42.55	42.55±14.77
Theaspirane	1523	1.76	0	0	0	3.75	3.75±2.48

Data from the same laboratory, adapted from Huimin Wu et al. (2022) [77], Cai Zhao et al. (2022) [78] and Yuanyuan Miao et al. (2022) [79]. The confidence interval and average values of 12 volatile compound concentrations in the 4 flat peaches from Xinjiang, including Qingpan (QP), Wanpan (WP), Ruipan 2 (R2) and Ruipan 4 (R4). Nectarine (*Prunus nucipersica L.*), yellow nectarine (YN); apricot (*Prunus armeniaca L.*), Dabaiyou (DBY); grape (*Vitis vinifera L.*), Cabernet Sauvignon (CS).

Supplementary Figures

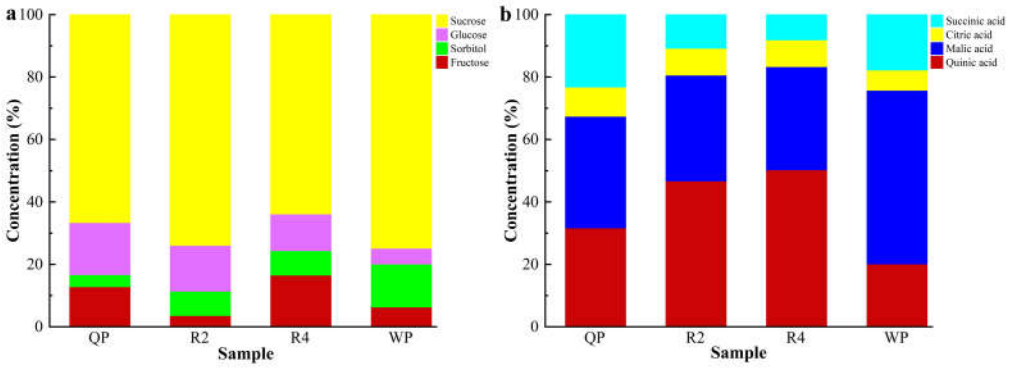

Figure S1. The concentration of soluble sugars and organic acids of flat peach in Xinjiang.

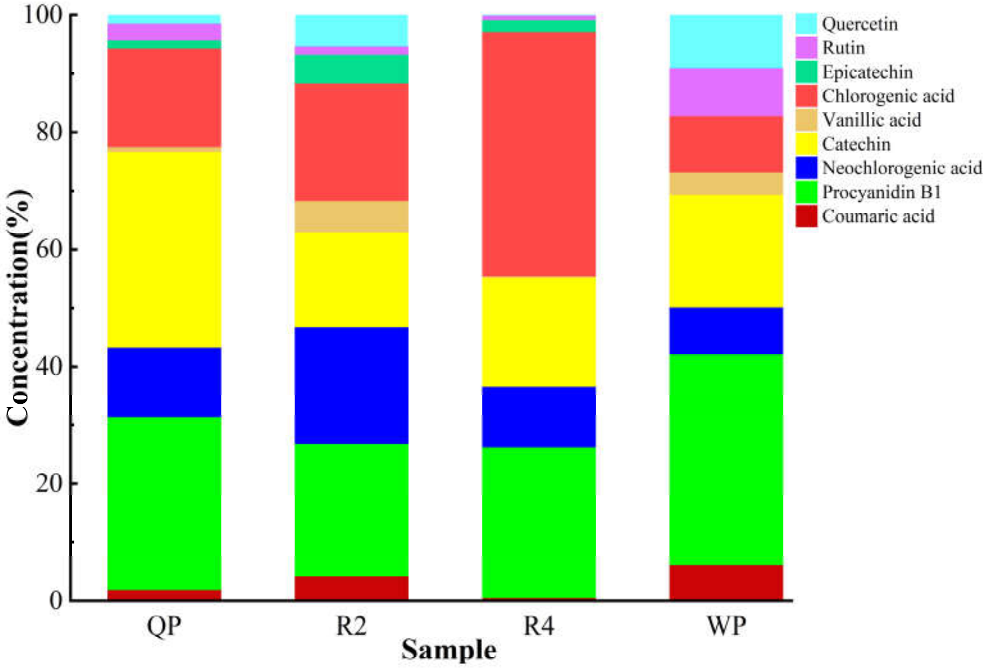

Figure S2. The concentration of phenolic compounds of flat peach in Xinjiang.

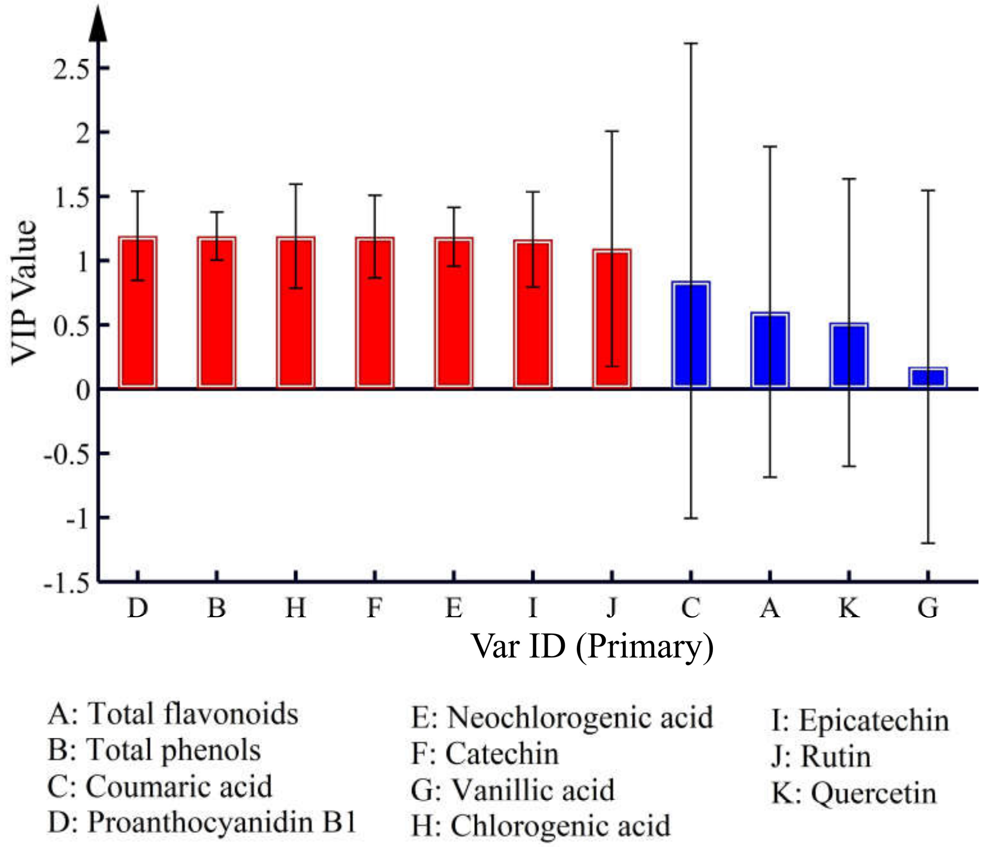

Figure S3. Importance of variables between phenolic compounds and antioxidant activity of flat peach in Xinjiang. Red means VIP more than 1, blue means VIP less than 1. The variable importance (VIP) value represents relevance.

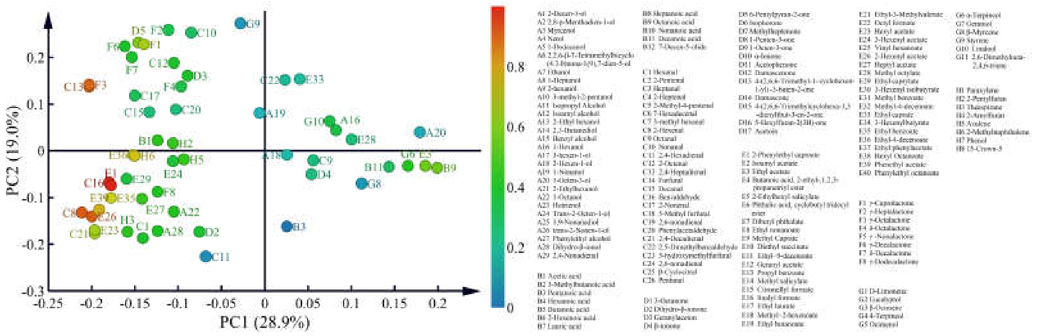

Figure S4. The loading diagram of PCA of volatile compounds of flat peach in Xinjiang.

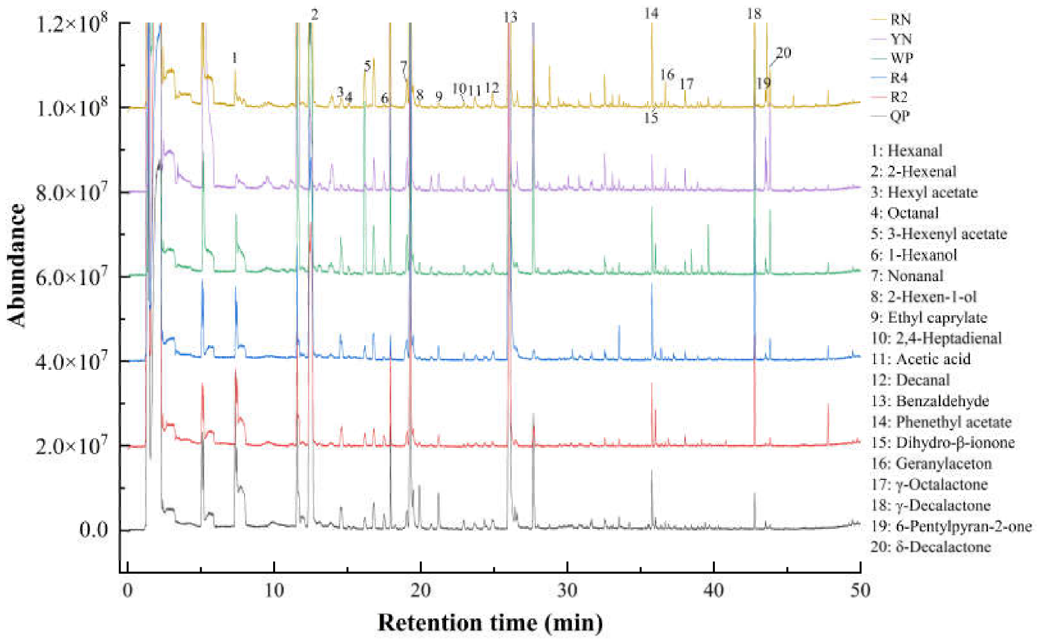

Figure S5. Overlay of the common ion chromatograms in the HS-SPME-GC-MS analysis of flat peaches and nectarines. Flat peaches, including Qingpan (QP), Wanpan (WP), Ruipan 2 (R2) and Ruipan 4 (R4); nectarines (*Prunus nucipersica L.*), including yellow nectarine (YN) and red nectarine (RN). The data of nectarines from the same laboratory, adapted from Huimin Wu et al. (2022).

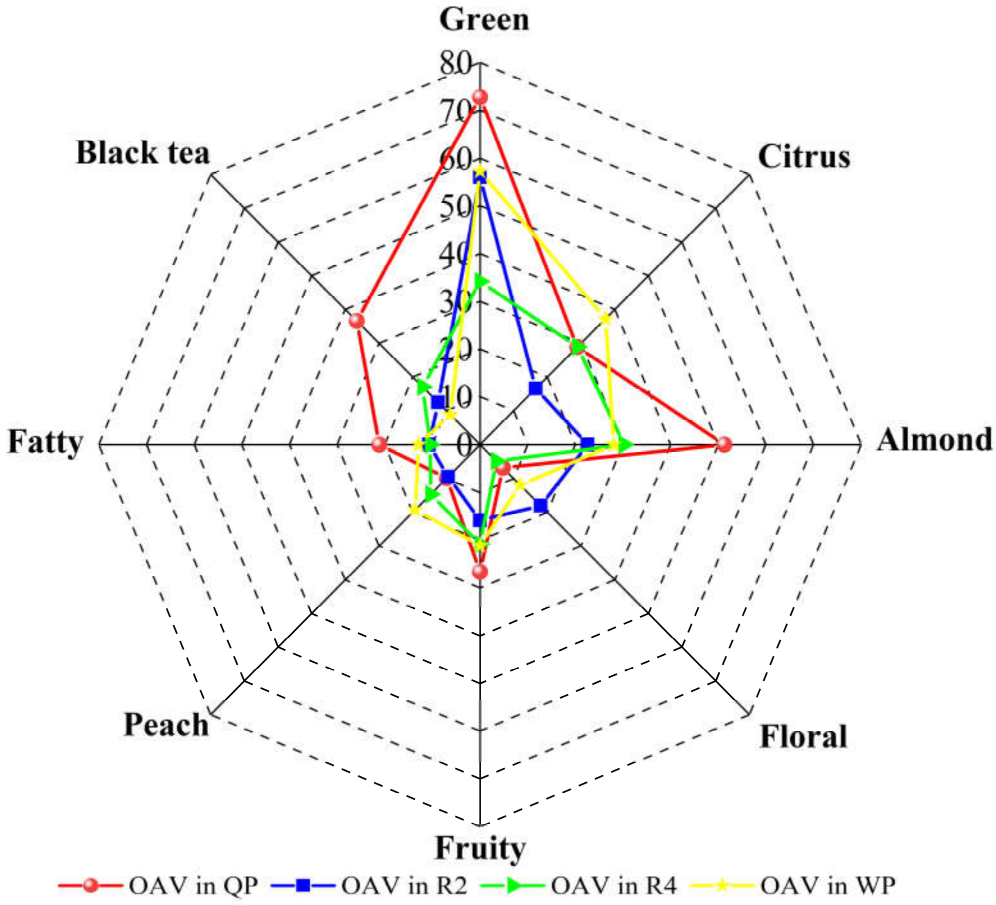

Figure S6. Odor characteristics of main aroma substances of flat peach in Xinjiang.
